# Design and evaluation of a custom circulating tumour DNA assay to detect endometrial cancer recurrence

**DOI:** 10.1038/s41698-025-01246-4

**Published:** 2026-03-23

**Authors:** M. Wadsley, DS Guttery, C. Cowley, G. Donaldson, C. Moreman, R. Hew, E. Stannard, L. Zhang, A. Collins, J. Shaw, Moss EL

**Affiliations:** 1 Leicester Cancer Research Centre, College of Life Sciences, University of Leicester, Leicester, UK; 2 University Hospitals of Leicester NHS Trust, Infirmary Square, Leicester, UK

**Keywords:** Biomarkers, Cancer, Genetics, Oncology

## Abstract

Circulating tumour DNA (ctDNA) has high sensitivity to detect endometrial cancer (EC) recurrence. An EC-specific ctDNA panel (ECctDNA-panel) was designed using TCGA/CPTAC mutation profile datasets and whole exome sequencing data from primary ECs. The ECctDNA-panel was tested using commercial standards to determine the detection limit for known driver variants, before investigating the plasma cell free DNA (cfDNA) from patients with/without recurrence. The ECctDNA-panel was able to detect EC hotspot mutations at >1% AF in 100% (42/42) of primary tumours tested. The ctDNA standards confirmed detection as low as 0.74% VAF in 5 ng template DNA. The ECctDNA-panel detected hotspot variants in 10/14 patients with recurrence and in 1/25 without recurrence: sensitivity/specificity 71.4%/96% and accuracy 87.2%. Potentially actionable mutations were identified in 8/10 ctDNA positive recurrences. We report the development of an ECctDNA-panel that has a high diagnostic accuracy to detect EC recurrence and could be utilised to guide patient’s further management.

## Introduction

Endometrial cancer (EC) incidence is rising in many countries and it is now the commonest gynaecological cancer in the UK^1^. In the USA the prevalence increased from 757,190^2^ in 2016 to 945,540 in 2025^3^. The challenge of identifying patients with EC recurrence for timely intervention and second-line systemic treatment or cytoreductive surgery is compounded by increasing EC prevalence, due to good long term survival in many cases^1^. Risk stratification using the TCGA molecular classification provides prognostic information compared to clinical/pathological/stage information however, such classifications are not able to identify individual patients within these categories who at increased risk of recurrence. This is exemplified in the high-risk PORTEC3 population where less than 31% developed a recurrence within 5 years^4^. Intensive follow-up with clinical examination and imaging has not been shown to improve overall survival^5^, however asymptomatic diagnosis of EC recurrence is associated with a better long-term outcome compared with symptomatic presentation^6^. EC recurrence can occur many years after completion of primary treatment, especially for the low-grade endometrioid subtype^7^, when many patients would have been discharged from routine clinical follow-up, and where histopathological confirmation would be required to confirm primary origin of malignancy. Biopsy of a suspected recurrence is an invasive procedure that can be associated with morbidity and rarely mortality^8,9^, as well as slowing the patient’s diagnostic journey due to the need for pathological analysis.

Circulating tumour DNA (ctDNA) has been shown to be a highly sensitive/specific biomarker for detecting cancer recurrence in many different cancers^10^, including EC^11–14^. A negative ctDNA test at any time following cancer treatment is highly predictive of no cancer recurrence, hazard ratio 35.8, *p* < 0.001^15^. A systematic review of blood-based biomarkers identified ctDNA as having superior diagnostic ability for detecting EC recurrence compared to other currently available biomarkers (CA125/HE4)^16^. Various ctDNA detection methods have been explored in EC including personalised assays, off the shelf panels, including Guardant360^17^, Oncomine^18^ and Imagia Canexia Health panels^19^, and mutation specific ddPCR^11^. Standardised, validated ctDNA panels have advantages over personalised assays since they do not require sequencing of the patient’s primary tumour and do not require in-depth bioinformatic analysis, therefore can give a result in a shorter clinically meaningful time period. The ability of panels to give information that can guide patient therapy is also an advantage, in particular identifying actional mutations and micro-satellite instability^20,21^, enabling the identification of potential systematic therapies.

The aim of this study was to design and evaluate an EC-specific ctDNA (ECctDNA) panel covering all the molecular EC subtypes, and including information required for molecular classification, mis-match repair gene status, TP53, and POLE applicable for use in a semi-automated workflow in a diagnostic service laboratory. To this end, the assay was set up and run on the ThermoFisher Ion Torrent Genexus System (next generation sequencing platform) using an automated nucleic acid to data analysis workflow in an ISO:15189:2022 accredited laboratory. The proposed indication for the ECctDNA panel was as a diagnostic tool to improve the detection and diagnosis of EC recurrence.

## Results

In total samples from 42 patients with endometrial cancer who were recruited to the study and were followed up with blood sampling were included in the analysis (Table 1). Three patients (cases NU1-3) had blood samples taken at the time of investigation for suspected endometrial cancer recurrence, however, were diagnosed with a second non-endometrial malignancy, breast cancer in two cases and in one case lymphoma. Of the remaining 39 cases, 14 (cases R1-14) had a sample for ctDNA analysis taken within 4 months of a clinical diagnosis of endometrial cancer recurrent/progressive disease.

**Table 1:** Demographic and tumour characteristics of patientswith and without an endometrial cancer recurrence (*n* = 39)

	Endometrial cancer recurrence (*n* = 14)	No endometrial cancer recurrence (*n* = 25)
**Median age at diagnosis**	70.5 years	62 years
(range)	(41–92 years)	(35–83 years)
**Histological subtype**		
Endometrioid	8	15
Serous	1	2
Clear cell	1	3
Carcinosarcoma	1	3
Mixed	3	2
**Tumour grade**		
Grade 1	4	7
Grade 2	2	5
Grade 3	8	13
**Stage at diagnosis**		
Stage I	6	14
Stage II	1	6
Stage III	4	5
Stage IV	3	0

Twenty-five patients (cases NR1-25) had a blood sample taken at a median of 60 months following diagnosis, and did not have a recurrence after a median of 82.5 months (range 59.0–122.5 months) follow-up (Table 1). There were no significant differences in the median age at diagnosis (p = 0.178), histological subtype (*p* = 0.862), tumour grade (*p* = 0.757) or stage at diagnosis (*p* = 0.431) between the recurrence and non-recurrence cases. Median endometrial cancer specific survival in patients with a confirmed recurrence was 39.3 months (range 0.2–147.5 months). At the study census date of 25/02/2025, four patients were alive following treatment for recurrence, two patients had undergone surgical excision, and two had received chemotherapy/radiotherapy treatment.

### Panel design and evaluation

The ECctDNA-panel design was based on variants observed in over 600 cancers within the TCGA and CPTAC datasets, in addition to variants from the sequenced WES data from 46 primary FFPE tumour DNA samples. The final design included 635-amplicons in total covering 1848 known hotspots, with 30 hotspots derived from WES data and known germline drivers, and, also included five clinically relevant MSI markers (MONO27, NR21, NR22, NR24, NR27). The panel was compared to gene lists from larger commercially available pan-cancer assays to ensure inclusion of key variant hotspots with relevance to EC.

### FFPE primary tumour DNA exome analysis

All 42 FFPE tumour DNA samples were analysed by WES and the ECctDNA-panel (Supplementary data: Table 1). Fifty-eight of the variants called in exome analysis were covered by the panel. Fifty of these variants were detected in FFPE DNA of the same samples using the panel. The eight missing variants were due to either sequencing or sample quality issues. Three of these missing variants, from cases NR4 and R10, were due to poor sample quality. The variant in case NR20 was present but below the quality threshold, likely due to this sample having a lower DNA input (7 ng). PTEN K267fs in case NR9, KMT2D p.P647fs in case NR11 were poorly covered in these samples with insufficient molecular reads to reliably call variants at these loci. KMT2D V3089fs in NR18 was present at 21% but was filtered out by Torrent Suite as a strand specific error. In case NR24, two different FFPE tumour cores were used for the exome analysis and the panel sequencing from the same FFPE block due to insufficient DNA yield from the first DNA extraction, which may explain the absence of PTEN R130G in the panel results. Of the 42 FFPE DNA samples sequenced with the panel, 39 had hotspot variants detected above the 5% AF threshold. The 3 remaining samples had hotspot variants detected but below the threshold at ~2.5% AF. No other variants of note were detected when no filter was applied (Supplementary data: Table 2).

### Limit of detection using reference standards

The ECctDNA-panel was able to pick up 16/17 known variants at 20 ng input DNA and 15/17 at 5 ng input DNA using the Horizon Oncospan cfDNA HD833 reference. Missing variants were deletions and not called by the Torrent Variant Caller with default settings but, were present on visual review in Interactive Genome Viewer (IGV). The Horizon HD842 standard for five low frequency variants ranging in variant allele frequency (VAF) from 1.88 to 8% showed the panel detected 100% of variants using both 5 ng and 10 ng input DNA. In addition, a 1:10 dilution of 5 ng of the HD842 was able to detect one of five variants at 0.74% VAF, with two other variants present but below quality score threshold at frequencies of 0.6% and 0.4% VAF (Supplementary data: Table 3).

### Panel testing in patient cfDNA samples

**Fig. 1:**
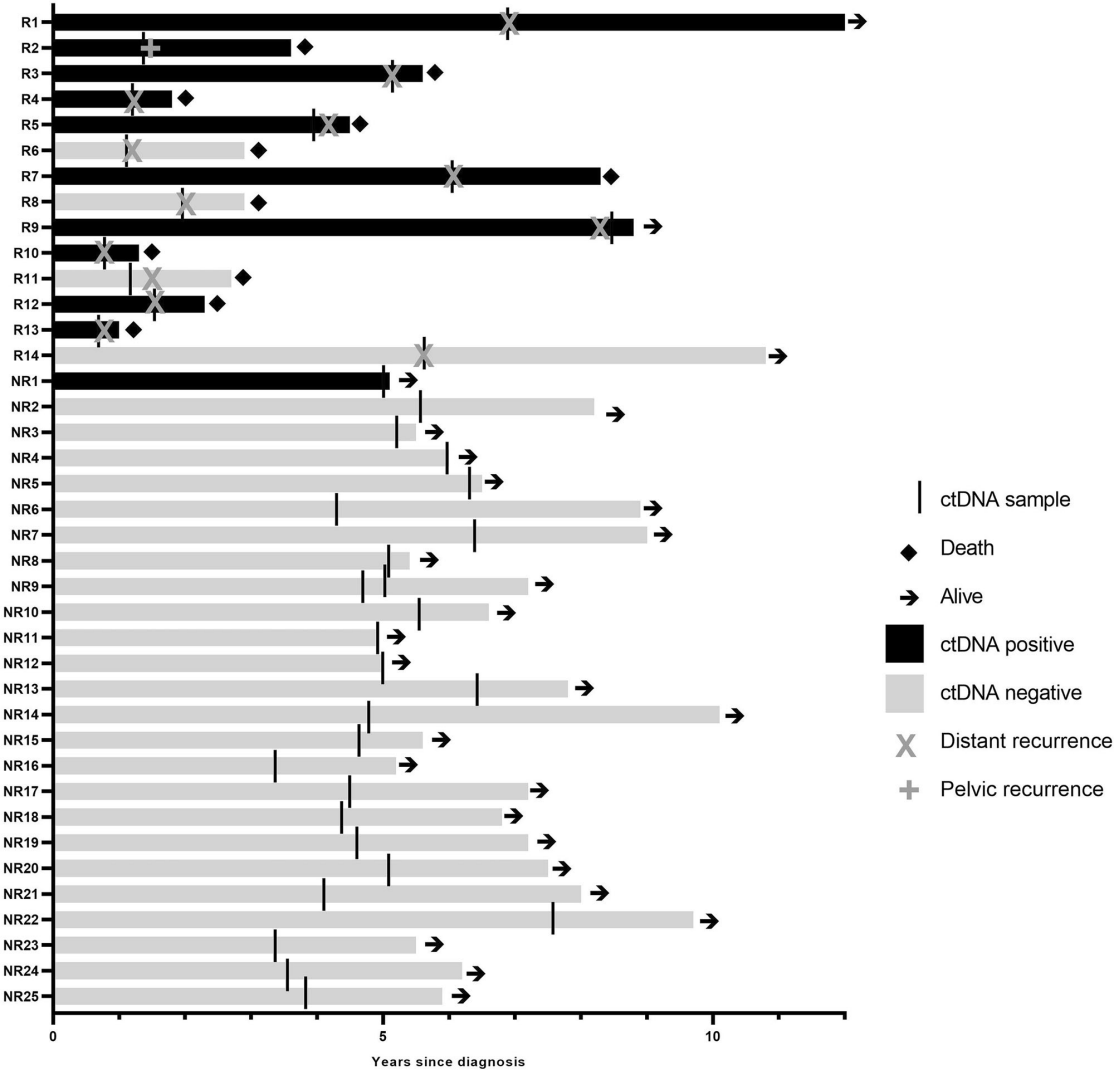
Timing of ctDNA sample and patient outcomes. R recurrence (*n* = 14), NR no recurrence (*n* = 25).

cfDNA from the 25 non-recurrences (NR1-25) and 14 recurrences (R1-14) was tested using the ECctDNA panel (Fig. 1 and Supplementary data: Tables 4 and 5).

All samples were run from sample to reportable results within a 48-h period at an approximate cost of £1000 per sample. Of the 635 amplicons, two failed in all cfDNA samples (a PMS2 variant and MONO27), and an additional 14 amplicons had a median functional molecular coverage below the variant calling threshold of 2 and so were excluded from subsequent analysis.

**Fig. 2:**
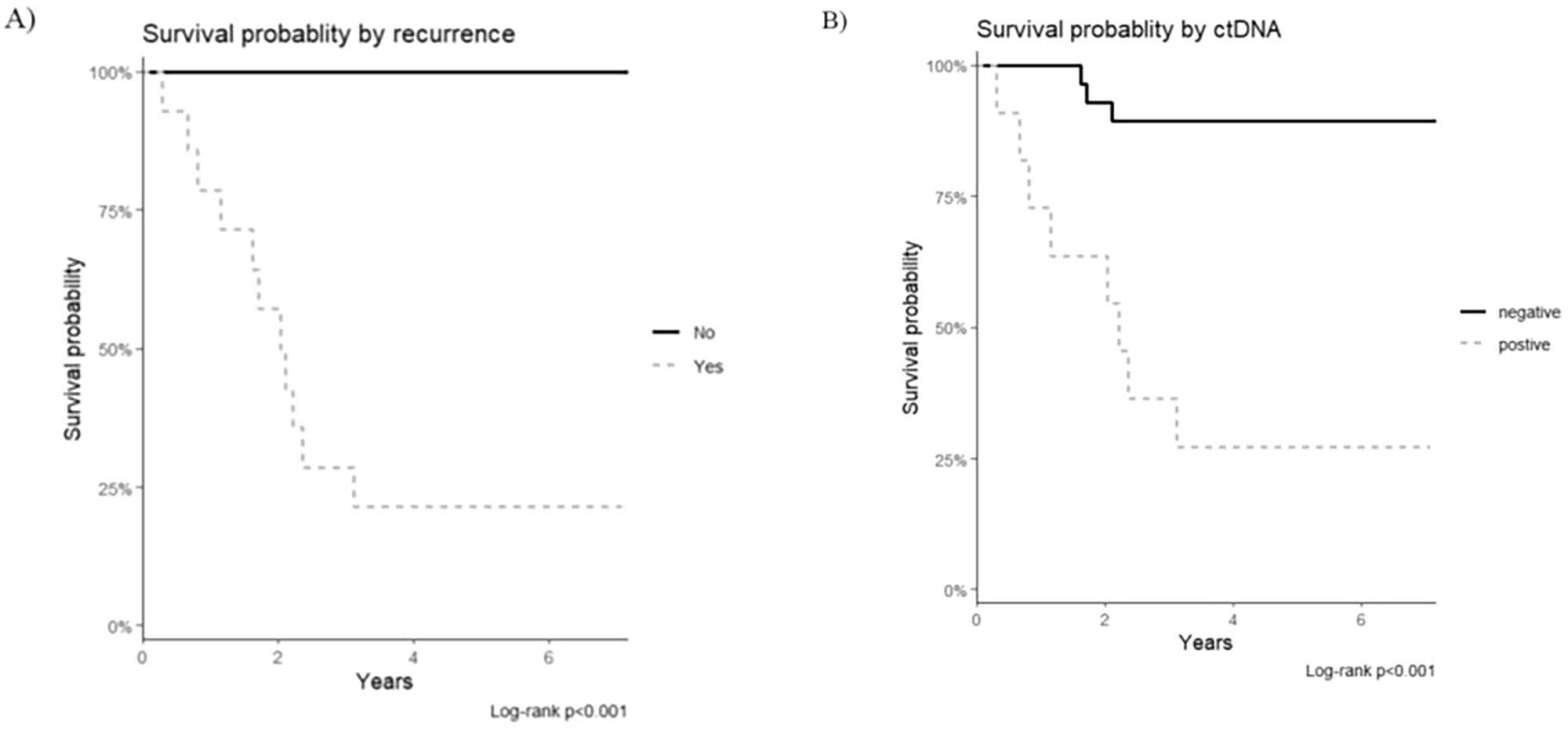
Overall survival of the study population. **A** Survival by endometrial cancer recurrence (Yes; *n* = 14) and no recurrence (No; *n* = 25). **B** Survival by positive ctDNA result (*n* = 11) and negative ctDNA result (*n* = 28).

Somatic hotspot variants were detected in cfDNA of 10 of the 14 recurrences and 1 of 25 non-recurrences (Supplementary data: Table 1). Potentially actionable variants were identified in 8 of the 10 recurrence cases with detectable ctDNA (Supplementary data: Table 6). Two cases with a non-uterine recurrence had germline variants detected that are known to be pathogenic for increased cancer risk. There was no significant difference in the quantity of total cfDNA analysed between the true positive and false negative cases median 15.5 ng (range 7–20 ng) and median 13.5 ng (range 5–20 ng). The median survival in patients with a positive ctDNA result was 36.1 months (Fig. 2).

Four of the fourteen cases with recurrence had no somatic variants detected by the ECctDNA- panel in cfDNA. In two of these cases (R6 and R11) a PPP2R1A p.P179R variant was detected in the tumour DNA (Supplementary data: Table 1). Reads supporting this variant were present in both cfDNA samples of these cases but did not meet quality thresholds to call. Case R14 had two hotspot variants, CTCF p.T204fs and CTNNB1 p.T41I detected in the tumour DNA exome and panel analysis but none were present in cfDNA despite amplicons covering these regions being at high depth. Case R8 had a PIK3CA E545K at 15% AF in tumour but was not detected in cfDNA. In addition to somatic hotspots, a heterozygous germline CHEK2 T367Mfs variant, and a heterozygous MLH1 C680R variant were identified in two separate cases, both of which are associated with increased cancer risk.

Although the amplicons designed to cover five MSI markers worked well with genomic DNA derived from buffy coat samples, the sequencing was more variable in depth for cfDNA samples. In cfDNA, the MONO27 amplicon had zero functional molecular depth in all 42 samples, NR21 in 35/42, NR22 in 20/42, 21/42 in NR24 and 15/42 in NR27. MSI analysis of these markers using paired normal and cfDNA was therefore challenging due to this inconsistency in read depth.

### Specificity of the panel for EC

Three patients within the ECctDNA study had presented with disseminated malignancy during the study, however, further investigations confirmed a second primary as the cause, in two cases breast cancer, and one lymphoma. Analysis of ctDNA using the ECctDNA panel taken at the time of suspected recurrence was negative in two patients. One case had a TP53 variant in cfDNA that was likely from their second primary since this fitted with their clinical findings, was not detected on immunohistochemistry of the primary tumour, in the EC tumour DNA in exome or on panel testing, however sampling bias due to tumour heterogeneity within the endometrial cancer could also be an explanation.

## Discussion

We have designed and evaluated the utility of an EC-specific ctDNA panel (ECctDNA-panel) that can be used in everyday clinical practice to diagnose recurrence and guide personalised management. The panel was designed using the ThermoFisher White Glove service using their Ampliseq HD technology capable of detecting variants down to a low VAF (typically 0.1% from 20 ng cfDNA input) and therefore in a range appropriate for detection of low level of ctDNA, as might be expected in detection of molecular relapse/recurrence^22^. The panel testing of patient samples was carried out on the Ion Torrent Genexus System, in a quality managed laboratory facility, given the aim of developing a panel that could be utilised within the NHS, and our study sought to explore the feasibility of utilising the Ion Torrent Genexus System’s automated workflow for EC-specific ctDNA testing.

In the panel design we sought to create a pragmatic solution that was EC-specific, cost effective and had minimal sample input requirements. Similarly sized commercially available panels tend not to cover all EC-specific regions identified in our variant analysis. Existing commercially available panels that cover the required gene regions for EC are much larger pan-cancer panels and are associated with increased sequencing costs and sample input requirements (>20 ng), to ensure coverage of all targets. These pan-cancer panels have the advantage of a wide range of variants from many tumour sites, whereas in the case of follow-up for a single malignancy much of the panel would be redundant, supporting a role for a smaller, and therefore less financially costly, tumour-specific panel^23^. The potential impact on patient care also needs to be considered since a larger gene panel has been shown to identify a greater number of molecular-based recommended therapies compared to a more limited gene panel, however this has not been shown to result into improved patient outcomes^24^.

In the comparison with reference standards for cfDNA, the Horizon Oncospan cfDNA (HD833) showed consistent detection of a known variant down to 0.74% VAF using 5 ng cfDNA. Given that variants below this threshold may potentially be missed by the panel, we also reviewed variants called in cfDNA samples. The lowest frequency variants called were 0.75% VAF, which suggests that low frequency variants would be detected, however since VAF correlates with tumour volume^25^, very low volume recurrences could be below the limit of the ECctDNA panel’s detection. Detection of very low volume endometrial cancer recurrences that are not visible on imaging raises questions regarding patient management, since the impact of systematic therapy on long-term prognosis in such cases has not been investigated. Indeed, the OVO5 study did not show a survival benefit in ovarian cancer for early chemotherapy treatment based on biochemical relapse using the tumour marker CA125^26^. Patients have raised concerns regarding the psychological impact of a long lead time between ctDNA and clinical relapse^27^ and therefore having a panel that is not able to detect ultra-low volume disease, rather instead diagnose measurable recurrence with a view to instigating treatment may be more clinically relevant at this time.

In cfDNA samples the panel performed well with only 2 out of 635 amplicons failing to sequence, a median base coverage of 28825.5X and a median molecular coverage of 452X across all samples. Inconsistent coverage of the MSI markers in cfDNA samples made MSI analysis difficult and refinements in panel design will be made to ensure effective MSI detection in a future version of the panel. Given the introduction of universal tissue testing for mismatch repair (MMR) deficiency at EC diagnosis and the inclusion of immune checkpoint inhibitors for MMR proficient tumours^28^, the inclusion of MSI markers in the panel may not add additional information to guide patients’ subsequent management. Other variants in homopolymer regions were also challenging to call. CTCF frameshift variants (CTCF p.T204fs) frequently observed in EC^20^ were inconsistently called in our cohort with 20 of the FFPE samples having calls at this site with only 8 observed in exome data, and none in cfDNA. Better resolution of calls at this locus could have led to a positive identification of recurrence in case R14. As part of the planned extension of this study sequencing of further samples will allow refinement of calling in these regions to enhance variant detection and MSI calling.

Across the 42 patient samples, there was a single false positive result in the 25 patients without a recurrence. The variant observed in the false positive case was ARID1A p.R1109Q, which has been observed once in TCGA for EC but is a predicted passenger variant and has no association in the literature with EC, and the patient has remained disease-free. In two of the patient samples, germline variants included on the panel as potential germline drivers were detected. One of these patients was a non-EC recurrence at time of sequencing but subsequently developed a breast cancer, demonstrating the value of identifying these variants on the panel for closer monitoring of patients. Ten of 14 recurrence cases were detected through ctDNA, 8 with actionable mutations that could guide treatment at the time of recurrence. Of note three of the four recurrences without hotspots detected in ctDNA had one or more low frequency variants detected by sequencing that failed to meet thresholds for variant calling, but could herald a request for a repeat test. As more samples are processed through the panel a sequence variant baseline will be built that may allow better resolution of these low frequency variants. Although there were no Clonal Hematopoiesis of Indeterminate Potential (CHIP) mutations detected in these patients, future studies could address CHIP through comparison with paired buffy coat DNA form the same blood sample.

The ECctDNA-panel could also be used for resolving diagnostic dilemmas in patients, potentially negating the need for a diagnostic biopsy. ctDNA has been shown to have greater diagnostic ability for EC recurrence compared to other available blood-based biomarkers with a sensitivity of 0.87 (95% CI: 0.63, 0.99) and specificity of 0.89 (95% CI: 0.70, 0.98), versus 0.49 (95% CI: 0.28, 0.70) and 0.91 (95% CI: 0.77, 0.97) for CA125, and 0.74 (0.64, 0.82) and 0.71 (95% CI: 0.40, 0.91) for HE4^16^. Therefore, if mutations identified by the ECctDNA-panel in ctDNA were compared to those detected in the primary cancer or common endometrial cancer associated mutations it could either confirm EC recurrence, or, where independent only mutations were detected, a second primary cancer, as illustrated by NU1, where the TP53 mutation detected on ctDNA was not present in the primary EC but was in-keeping with the patient’s breast cancer diagnosis. ctDNA testing could facilitate the diagnosis of EC recurrence, enabling more timely diagnosis and avoiding the need for invasive biopsies, as well as guiding further oncological treatment due to the identification of actionable mutations^29^. Furthermore, as plasma and tumour samples were collected at different time points the emergence of novel mutations over time, identified through longitudinal blood sampling can aid in profiling potential tumour evolution.

Given the financial cost of clinical follow-up and investigating/confirming a cancer recurrence^30^, currently achieved through clinical examination, imaging and an interventional radiology or surgical biopsy, the potential of a blood test giving a definitive diagnosis within 48-h could have a significant impact. Clinicians involved in the care of EC patients were supportive of a biomarker with high sensitivity and specificity that could be used to detect recurrence since it was felt would it could increase the confidence of clinicians and patients in reduced face-to-face clinical follow-up strategies, for example patient-initiative follow-up^31,32^. In addition, many patients experience significant negative physical and psychological sequalae following EC treatment, with pelvic examination often cited as a re-traumatising or painful experience^33^. The potential for an EC follow-up scheme based on a blood-test rather than clinical examination has strong patient support^27^ and would enable clinicians to develop more comprehensive trauma-informed care strategies^34^ for patients where clinical examination is not possible, for example following exenterative surgery, or not acceptable, including survivors of sexual trauma.

Further testing of this ECctDNA panel is planned in a larger cohort of recurrent cases, which will enable minor refinement of the panel in order to remove redundant markers and redesign the few markers that did not perform as well, and will increase the robustness of the results.

The limitations of this study include the small sample numbers, however the use of data from the TCGA and CPTAC databases has enabled information from a larger, geographically and ethnically diverse population to be considered in the design of the panel, therefore increasing its potential utility in other populations^35^. Although there were only 14 recurrences within the study population, cases included all the histological subtypes and grades thereby supporting the utility of the ECctDNA in all endometrial cancers, not just high-risk cases with the greater risk of recurrence which has been the focus previously for ctDNA studies^36^. The timing of the samples was pragmatic in order not to include the burden of appointments for patients, however Covid-19 restrictions did impact on patient follow-up appointments resulting in patients without a cancer recurrence having blood samples towards the end of their 5-year follow-up period.

We report the development and evaluation of an EC-specific ctDNA-panel that has a high diagnostic accuracy to detect EC recurrence and could be utilised to guide patient’s further management through information of potential actionable variants.

## Methods

The study was conducted in accordance with the Declaration of Helsinki and approved by the Wales 7 Research Ethics Committee (17/WA/034). Patients under follow-up for an endometrial cancer were recruited between December 2017 and December 2022. All participants gave written informed consent. Demographic and clinicopathological information were collected for each participant and a blood sample taken at each follow-up appointment. Participants continued in the study until the end of the study, they wished to withdraw, completed clinical follow-up, or until they died of disease. During the Covid-19 pandemic, many face-to-face follow-up appointments were changed to telephone appointments, which inhibited the ability to collect follow up blood samples from patients without recurrence. Up to 20 mL of blood was collected in K2 EDTA tubes (BD Biosciences) and processed to plasma and buffy coat within 2 h of collection as described previously^37^. FFPE tumour tissue blocks were retrieved from the pathology archive for retrieval of tumour cells.

### Extraction and quantitation of DNA

Total cfDNA was isolated from 4 mL of plasma with the MagMAX Cell-free DNA Isolation Kit (Thermo Fisher Scientific) according to manufacturer’s instructions, and Genomic DNA (gDNA) was isolated from 200 µl buffy coat using the DNA blood mini kit (Qiagen) as per manufacturer’s instructions. FFPE tumour DNA from 1-mm tissue FFPE cores were taken after review by a consultant histopathologist and DNA was extracted manually using the Qiagen GeneRead Kit (Qiagen) or semiautomated using the MagMAX™ FFPE DNA/RNA Ultra Kit on the Kingfisher Flex (Thermo Fisher Scientific) as per manufacturer’s instructions. In cases where the tumour material was limited, DNA was recovered from more than once area (core) and mixed in equal ratios prior to sequencing, aiming to mitigate limiting analysis to a single tumour area and reduce the impact of tumour heterogeneity. Quantitation and integrity checks were performed using the Qubit dsDNA BR Assay Kit (Thermo Fisher Scientific) on the Qubit 4.0 fluorometer as per manufacturer’s instructions and on the Agilent TapeStation 4200 using the HS D5000 assay (Agilent) and the Cell-Free DNA assay (Agilent) according to manufacturer’s instructions.

### Whole exome sequencing and analysis

50 ng of tumour DNA and matched buffy coat DNA underwent whole exome sequencing (WES) using the Illumina NovaSeq 6000 (Novogene). The minimum average depth of coverage was 50x for buffy coat samples and 200x for tumour samples. In one case no primary tumour was available, and instead tissue from a resected recurrence was sequenced.

Adapter trimming of FASTQs was done using trimmomatic 0.36 and reads were aligned to the hg38 human reference using bwa 0.7.17. Picard 2.6.0 was used to mark duplicate reads for downstream analysis. BQSR was done as part of GATK 4.3.0 and a panel of normals was generated using Mutect2 in tumour-only mode on the 46 normal samples. NGS-Checkmate was performed on all bams as an identity check prior to paired analysis.

Mutect2 was used to call somatic variants. SNV variants were filtered out if overall depth <30, alt allele depth <5, alt allele was present in the normal or allele frequency was <1%. Indel variants were filtered out if overall depth <50, alt allele depth <10, alt allele was present in the normal or allele frequency was <1%. Variants were annotated with Funcotator in GATK4.3.0 including annotation from the Cancer Gene Census v99. MSI analysis was performed on exome samples using MSI-sensor2 (https://github.com/niu-lab/msisensor2).

### Design of an ECctDNA ion Ampliseq HD panel

Firstly, an EC-specific ctDNA panel was designed to incorporate the somatic single nucleotide variants identified in the TCGA (endometrial cancer and carcinosarcoma) and CPTAC datasets, along with known germline driver variants for endometrial cancer^38^. Variants present in more than three patients were considered for inclusion on the panel. A custom Ampliseq-HD DNA hotspot panel was designed using the ThermoFisher White Glove team covering all essential variants and MSI targets.

Secondly, the coverage of the designed panel was compared to the tumour variants from the whole exome data of the Leicester patient cohort (*n* = 42). Additional variants were considered for inclusion if they were present in more than 2/42 patients and were at >5% allele frequency. The panel underwent revision to include 30 additional variants and the removal of redundant MSI markers to form the final panel for ctDNA testing. Gene regions in the panel were compared to genes present in commercially available ctDNA panels.

### Target NGS and analysis

Targeted next-generation sequencing (NGS) was conducted using 10 ng FFPE tumour tissue DNA, 10 ng of gDNA and a median of 16 ng cfDNA (range 5–20ng) using the Ion Ampliseq HD customised panel (Thermo Fisher Scientific) on the automated workflow ‘nucleic acid to data analysis’ on the Ion Torrent Genexus (Thermo Fisher Scientific) as per manufacturer’s instructions. Alignment of sequencing raw data (hg19) and variant calling was performed by the Torrent Suite Software version v6.8.2.0 using a custom workflow set up as instructed in the Custom Assay Guide (MAN0028005 revision:A.0). Variants were filtered to loci present in a submitted hotspot bed file. Additional filtering was performed based on presence of the variant in >=3 molecular families and an allele frequency of >=5% for FFPE and any variant in >=2 molecular families for cfDNA. Actionable mutations were identified using OncoKb^39,40^. Panel testing was conducted on (1) DNA from tumour tissue; (2) EC plasma cfDNA samples; and (3) commercial ctDNA reference standards (HD833 & HD842, Horizon Discovery) with known variants and limit of detection determined using a range of DNA input.

### Statistical analysis

Statistical analysis was performed using the R statistical language (v4.3.1)^41^. Sensitivity and Specificity calculations were generated using the epiR package (v2.0.84).

## Supplementary information

Supplementary data

## Data Availability

Data used were either publicly available or from patients. Deidentified reconstructed data for individual patients is available in the supplementary data.
