# Supplementary material for: Design and evaluation of a custom circulating tumour DNA assay to detect endometrial cancer recurrence
Source: NPJ Precis Oncol. 2026 Mar 23;10:127. doi: 10.1038/s41698-025-01246-4 (PMC13009394; doi:10.1038/s41698-025-01246-4)
Supplement: Supplementary file 1 — Supplementary data [file 41698_2025_1246_MOESM1_ESM.pdf]

Supplementary data

Key: EE – endometrioid; CS – carcinosarcoma; CCC – clear cell carcinoma; S – serous. R – Recurrence; NR – no recurrence; NU – not uterine cancer recurrence

Patient	Result_Type	Histology	Grade	Locus	Variant	Exome_AF	Exome_QUAL	Exome_Alt_Depth	Exome_Total_Depth	Tumour_AF	Tumour_QUAL	Tumour_Alt_Depth	Tumour_Total_Depth	cfDNA_AF	cfDNA_QUAL	cfDNA_Alt_Depth	cfDNA_Total_Depth	cfDNA variant label
R1	Recurrence	EE	1	chr3-178936074-C-G	PIK3CA_p.P539R	0.071	-	8	142	0.1368	321.435	32	234	0.0111	17.9504	12	1082	Somatic
R1	Recurrence	EE	1	chr3-178952085-A-G	PIK3CA_p.H1047R	0.058	-	11	182	0.1941	666.757	59	304	-	-	-	-	
R1	Recurrence	EE	1	chr3-41266100-T-C	CTNNB1_p.S33P	-	-	-	-	0.179	205.823	75	419	-	-	-	-	
R1	Recurrence	EE	1	chr3-41266103-G-C	CTNNB1_p.G34R	-	-	-	-	0.1874	230.742	80	427	-	-	-	-	
R1	Recurrence	EE	1	chr3-41266112-T-C	CTNNB1_p.S37P	-	-	-	-	0.186	195.693	80	430	-	-	-	-	
NR1	No Recurrence	EE	1	chr10-89720832-CAAAT-C	PTEN_p.N329KfsTer14	-	-	-	-	0.0556	73.4706	10	180	-	-	-	-	
NR1	No Recurrence	EE	1	chr1-27105930-TG-T	ARID1A_p.D1850TfsTer33	-	-	-	-	0.2727	19.7126	3	11	-	-	-	-	
NR1	No Recurrence	EE	1	chr3-178936091-G-A	PIK3CA_p.E545K	0.111	-	5	65	0.1136	139.268	15	132	-	-	-	-	
NR1	No Recurrence	EE	1	chr3-41266101-C-A	CTNNB1_p.S33Y	0.071	-	25	383	0.123	213.71	23	187	-	-	-	-	
NR1	No Recurrence	EE	1	chr1-27097737-G-A	ARID1A_p.R1109Q	-	-	-	-	-	-	-	-	0.0091	8.22743	2	220	Somatic
NR2	No Recurrence	EE	3	chr12-25398284-C-T	KRAS_p.G12D	-	-	-	-	0.1463	56.1845	6	41	-	-	-	-	
NR3	No Recurrence	EE	3	chr10-89717769-TA-T	PTEN_p.K267RfsTer9	-	-	-	-	0.3846	186.293	10	26	-	-	-	-	
NR3	No Recurrence	EE	3	chr3-178936096-G-T	PIK3CA_p.Q546H	0.142	-	6	43	0.2632	1611.46	125	475	-	-	-	-	
NR4	No Recurrence	EE	2	chr10-89692904-C-G	PTEN_p.R130G	0.071	-	6	126	-	-	-	-	-	-	-	-	
NR4	No Recurrence	EE	2	chrX-39921444-T-C	BCOR_p.N1425S	0.079	-	7	89	-	-	-	-	-	-	-	-	
R2	Recurrence	EE	1	chr10-123279677-G-C	FGFR2_p.S252W	NA	NA	NA	NA	0.3851	599.345	62	161	0.0132	17.7884	7	529	Somatic
R2	Recurrence	EE	1	chr10-89692905-G-A	PTEN_p.R130Q	NA	NA	NA	NA	0.8182	110.73	9	11	0.0287	58.64	13	453	Somatic
R2	Recurrence	EE	1	chr1-27101067-GC-G	ARID1A_p.Q1452RfsTer29	NA	NA	NA	NA	0.0847	44.6134	5	59	-	-	-	-	
R2	Recurrence	EE	1	chr1-27101116-AC-A	ARID1A_p.P1468LfsTer13	NA	NA	NA	NA	0.0877	45.1381	5	57	-	-	-	-	
R2	Recurrence	EE	1	chr4-153244184-C-T	FBXW7_p.R658Q	NA	NA	NA	NA	0.0732	25.1825	3	41	-	-	-	-	
NR5	No Recurrence	CS	3	chr10-89624245-G-T	PTEN_p.E7*	0.181	-	7	43	0.1805	261.566	24	133	-	-	-	-	
NR5	No Recurrence	CS	3	chr12-133212572-G-T	POLE_p.S1906Y	0.232	-	31	143	0.1538	256.882	24	156	-	-	-	-	
NR5	No Recurrence	CS	3	chr12-133253184-G-C	POLE_p.P286R	0.148	-	24	169	0.1606	317.108	31	193	-	-	-	-	
NR5	No Recurrence	CS	3	chr1-27105930-TG-T	ARID1A_p.D1850TfsTer33	-	-	-	-	0.625	32.9922	5	8	-	-	-	-	
NR5	No Recurrence	CS	3	chr1-27106354-C-T	ARID1A_p.R1772*,ARID1A_p.R1989*	0.054	-	11	234	0.0968	132.341	15	155	-	-	-	-	
NR5	No Recurrence	CS	3	chr17-7578212-G-A	TP53_p.R213*	0.362	-	101	287	0.3923	980.552	71	181	-	-	-	-	
NR5	No Recurrence	CS	3	chr2-48026752-G-T	MSH6_p.E544*	0.179	-	14	94	0.1006	158.326	18	179	-	-	-	-	
NR5	No Recurrence	CS	3	chr3-178916876-G-A	PIK3CA_p.R88Q	0.122	-	6	61	0.1644	134.912	12	73	-	-	-	-	
NR5	No Recurrence	CS	3	chr5-112175490-C-T	APC_p.S1400L	-	-	-	-	0.1307	200.823	20	153	-	-	-	-	
NR5	No Recurrence	CS	3	chr5-67589618-C-T	PIK3R1_p.R461*	-	-	-	-	0.2069	56.7963	6	29	-	-	-	-	
NR6	No Recurrence	CCC	3	chr12-25398284-C-T	KRAS_p.G12D	NA	NA	NA	NA	0.1007	125.748	14	139	-	-	-	-	
NR6	No Recurrence	CCC	3	chr3-178916876-G-A	PIK3CA_p.R88Q	NA	NA	NA	NA	0.1458	67.9715	7	48	-	-	-	-	
NR7	No Recurrence	CS	3	chr10-89717672-C-T	PTEN_p.R233*	0.241	-	44	179	0.2769	735.941	90	325	-	-	-	-	
NR7	No Recurrence	CS	3	chr1-27105930-TG-T	ARID1A_p.D1633fs	0.197	-	77	409	0.3	310.616	24	80	-	-	-	-	
NR7	No Recurrence	CS	3	chr22-29091856-AG-A	CHEK2_p.T367MfsTer15	0.759	-	22	29	0.4845	523.026	47	97	0.5357	3161.09	195	364	Germline
NR7	No Recurrence	CS	3	chr2-48026567-G-A	MSH6_p.R482Q	0.12	-	15	136	0.0827	46.494	11	133	-	-	-	-	
NR7	No Recurrence	CS	3	chr4-153249384-C-T	FBXW7_p.R465H,FBXW7_p.R385H	0.2	-	20	102	0.1891	294.659	45	238	-	-	-	-	
R3	Recurrence	EE	3	chr17-7578190-T-C	TP53_p.Y220C	NA	NA	NA	NA	0.2067	715.124	62	300	0.6002	10428.2	599	998	Somatic
NR8	No Recurrence	EE	1	chr3-178952085-A-G	PIK3CA_p.H1047R	0.188	-	34	197	0.1474	140.52	14	95	-	-	-	-	
NR9	No Recurrence	S	3	chr10-89692904-C-T	PTEN_p.R130*	0.197	-	48	246	0.2129	387.46	33	155	-	-	-	-	
NR9	No Recurrence	S	3	chr10-89717769-TA-T	PTEN_p.K267fs	0.202	-	30	149	-	-	-	-	-	-	-	-	
NR9	No Recurrence	S	3	chr1-27106861-C-T	ARID1A_p.R2158*,ARID1A_p.R1941*	0.179	-	99	589	0.1538	434.981	44	286	-	-	-	-	
NR9	No Recurrence	S	3	chr3-178916876-G-A	PIK3CA_p.R88Q	0.167	-	22	157	0.0833	44.6033	5	60	-	-	-	-	
NR9	No Recurrence	S	3	chr4-153249385-G-A	FBXW7_p.R465C,FBXW7_p.R385C	0.232	-	53	234	0.2367	585.684	49	207	-	-	-	-	
NR10	No Recurrence	EE	2	chr10-89711893-C-T	PTEN_p.Q171*	-	-	-	-	0.4	36.1858	4	10	-	-	-	-	
NR11	No Recurrence	CCC/EE	3	chr10-89720801-CTTACT-C	PTEN_p.T319NfsTer4	-	-	-	-	0.2691	85.2724	88	327	-	-	-	-	
NR11	No Recurrence	CCC/EE	3	chr12-49445525-TG-T	KMT2D_p.P647fs	0.32	-	37	109	-	-	-	-	-	-	-	-	
NR11	No Recurrence	CCC/EE	3	chr1-27105930-TG-T	ARID1A_p.D1633fs,ARID1A_p.D1850TfsTer33	0.382	-	268	692	0.3814	576.906	45	118	-	-	-	-	
NR11	No Recurrence	CCC/EE	3	chr3-178952072-A-G	PIK3CA_p.M1043V	0.4	-	111	273	0.3618	2770.41	195	539	-	-	-	-	
NR12	No Recurrence	EE	1	chr12-25398284-C-A	KRAS_p.G12V	-	-	-	-	0.3165	1707.22	125	395	-	-	-	-	
NR12	No Recurrence	EE	1	chr19-52715983-G-A	PPP2R1A_p.R183Q	-	-	-	-	0.3168	702.228	51	161	-	-	-	-	
NR13	No Recurrence	EE	1	chr10-123279677-G-C	FGFR2_p.S252W	-	-	-	-	0.0702	21.4348	4	57	-	-	-	-	
NR13	No Recurrence	EE	1	chr12-49433722-G-A	KMT2D_p.R2611C	-	-	-	-	0.0612	16.8852	3	49	-	-	-	-	
NR13	No Recurrence	EE	1	chr3-178936094-C-A	PIK3CA_p.Q546K	-	-	-	-	0.0684	64.7683	8	117	-	-	-	-	
NR13	No Recurrence	EE	1	chrX-39921444-T-C	BCOR_p.N1459S	-	-	-	-	0.0549	34.0399	13	237	-	-	-	-	
NR14	No Recurrence	EE	3	chr17-7577539-G-A	TP53_p.R248W	0.256	-	126	492	0.252	810.054	63	250	-	-	-	-	
NR14	No Recurrence	EE	3	chr3-178952085-A-T	PIK3CA_p.H1047L	0.427	-	107	229	0.4776	1467.39	96	201	-	-	-	-	
NR15	No Recurrence	EE	1	chr10-89624245-G-T	PTEN_p.E7*	0.184	-	10	53	0.137	296.116	30	219	-	-	-	-	
NR15	No Recurrence	EE	1	chr10-89711983-G-T	PTEN_p.E201*	-	-	-	-	0.1036	192.288	23	222	-	-	-	-	
NR15	No Recurrence	EE	1	chr12-133250289-C-A	POLE_p.V411L	0.119	-	8	76	0.1565	416.325	41	262	-	-	-	-	
NR15	No Recurrence	EE	1	chr1-27106354-C-T	ARID1A_p.R1772*,ARID1A_p.R1989*	0.264	-	62	232	0.2229	714.933	74	332	-	-	-	-	
NR15	No Recurrence	EE	1	chr16-67650710-C-T	CTCF_p.R339W	0.134	-	49	370	0.1412	457.093	48	340	-	-	-	-	
NR15	No Recurrence	EE	1	chr3-178916876-G-A	PIK3CA_p.R88Q	0.189	-	8	56	0.1321	213.683	21	159	-	-	-	-	
NR16	No Recurrence	EE	2	chr10-123258036-T-C	FGFR2_p.N321D,FGFR2_p.N549D	0.538	-	77	148	0.784	2342.58	167	213	-	-	-	-	
NR16	No Recurrence	EE	2	chr1-27105930-TG-T	ARID1A_p.D1850TfsTer33	-	-	-	-	0.4167	29.7949	5	12	-	-	-	-	
NR16	No Recurrence	EE	2	chr3-178952085-A-G	PIK3CA_p.H1047R	0.206	-	43	209	0.4655	794.536	54	116	-	-	-	-	
NR16	No Recurrence	EE	2	chr5-67589609-GA-G	PIK3R1_p.S460VfsTer20	-	-	-	-	0.4	27.6901	4	10	-	-	-	-	
NR17	No Recurrence	EE	1	chr10-89624275-C-T	PTEN_p.Q17*	0.375	-	67	185	0.3986	861.868	59	148	-	-	-	-	
NR17	No Recurrence	EE	1	chr3-178936094-C-A	PIK3CA_p.Q546K	0.125	-	8	61	0.1704	246.507	23	135	-	-	-	-	
R4	Recurrence	EE	3	chr10-89692905-G-T	PTEN_p.R130L	NA	NA	NA	NA	0.7654	2404.9	137	179	0.012	11.6361	3	250	Somatic
R5	Recurrence	EE	1	chr17-7577121-G-A	TP53_p.R273C	NA	NA	NA	NA	0.4259	2422.24	161	378	0.1647	729.832	70	425	Somatic
R5	Recurrence	EE	1	chr19-52715971-C-G	PPP2R1A_p.P179R	NA	NA	NA	NA	0.233	935.741	79	339	0.0694	246.777	34	490	Somatic
NR18	No Recurrence	CCC	3	chr10-89717769-TA-T	PTEN_p.K267RfsTer9,PTEN_p.K267fs	0.259	-	28	118	0.2623	198.248	16	61	-	-	-	-	
NR18	No Recurrence	CCC	3	chr12-49431873-AC-A	KMT2D_p.V3089fs	0.26	-	63	242	-	-	-	-	-	-	-	-	
NR18	No Recurrence	CCC	3	chr1-27105930-TG-T	ARID1A_p.D1633fs,ARID1A_p.D1850TfsTer33	0.243	-	141	600	0.3	310.616	24	80	-	-	-	-	
NR18	No Recurrence	CCC	3	chr19-52715982-C-T	PPP2R1A_p.R183W,PPP2R1A_p.R4W	0.23	-	28	129	0.2746	1365.85	106	386	-	-	-	-	
NR18	No Recurrence	CCC	3	chr3-37090443-T-C	MLH1_p.C680R	-	-	-	-	0.4675	1919.36	180	385	0.4932	2792.35	254	515	Germline
NR19	No Recurrence	S	3	chr10-89720677-T-A	PTEN_p.N276K	-	-	-	-	0.1456	210.229	38	261	-	-	-	-	
R6	Recurrence	S	3	chr19-52715971-C-G	PPP2R1A_p.P179R	NA	NA	NA	NA	0.7262	1014.08	61	84	-	-	-	-	
R7	Recurrence	EE	1	chr10-89692905-G-A	PTEN_p.R130Q	0.196	-	18	92	0.3077	35.6151	4	13	0.0147	37.9624	16	1087	Somatic
R7	Recurrence	EE	1	chr10-89717672-C-T	PTEN_p.R233*	-	-	-	-	0.1951	82.0234	8	41	0.026	126.358	36	1383	Somatic
R7	Recurrence	EE	1	chr12-133234521-G-A	POLE_p.T1104M	-	-	-	-	0.0638	25.1585	3	47	-	-	-	-	
R7	Recurrence	EE	1	chr12-25398284-C-G	KRAS_p.G12A	0.156	-	7	59	0.3396	233.905	18	53	0.0225	68.7691	21	935	Somatic
R7	Recurrence	EE	1	chr2-47705452-G-A	MSH2_p.G751E	-	-	-	-	0.075	25.1872	3	40	-	-	-	-	
R7	Recurrence	EE	1	chr3-178952085-A-T	PIK3CA_p.H1047L	-	-	-	-	0.1957	94.0935	9	46	0.0332	117.825	26	783	Somatic
R7	Recurrence	EE	1	chr3-41266124-A-G	CTNNB1_p.T41A	-	-	-	-	-	-	-	-	0.0241	97.0393	29	1201	Somatic
NR20	No Recurrence	CS	3	chr12-133257839-G-A	POLE_p.S30L	-	-	-	-	0.05	34.2619	4	80	-	-	-	-	
NR20	No Recurrence	CS	3	chr3-37042536-C-T	MLH1_p.R100*	-	-	-	-	0.2143	25.5669	3	14	-	-	-	-	
NR20	No Recurrence	CS	3	chr5-112173918-G-A	APC_p.R876Q	0.051	-	6	127	-	-	-	-	-	-	-	-	
NR20	No Recurrence	CS	3	chr5-67591106-A-G	PIK3R1_p.K567E,PIK3R1_p.K204E	0.425	-	32	76	0.5161	207.2	16	31	-	-	-	-	
R8	Recurrence	CCC/S	3	chr3-178936091-G-A	PIK3CA_p.E545K	NA	NA	NA	NA	0.15	25.3848	3	20	-	-	-	-	
NR21	No Recurrence	S/EE	3	chr19-52715971-C-G	PPP2R1A_p.P179R	-	-	-	-	0.3333	1240.54	92	276	-	-	-	-	
R9	Recurrence	EE	2	chr12-25380275-T-G	KRAS_p.Q61H	NA	NA	NA	NA	0.8282	10040	511	617	-	-	-	-	
R9	Recurrence	EE	2	chr4-153258982-C-T	FBXW7_p.R278Q	NA	NA	NA	NA	-	-	-	-	0.0075	6.54351	2	268	Somatic
NR22	No Recurrence	CS/S	3	chr3-178952006-T-C	PIK3CA_p.Y1021H	0.208	-	52	250	0.2423	1173.35	95	392	-	-	-	-	
NR22	No Recurrence	CS/S	3	chr3-178952072-A-G	PIK3CA_p.M1043V	0.181	-	39	222	0.2453	1273.07	105	428	-	-	-	-	
NR23	No Recurrence	EE	2	chr10-89692905-G-A	PTEN_p.R130Q	0.117	-	13	124	0.1677	811.329	80	477	-	-	-	-	
NR23	No Recurrence	EE	2	chr12-25398284-C-A	KRAS_p.G12V	-	-	-	-	0.1471	791.951	79	537	-	-	-	-	
NR23	No Recurrence	EE	2	chr12-49434491-AG-A	KMT2D_p.P2354LfsTer30	-	-	-	-	0.1622	56.3415	6	37	-	-	-	-	
R10	Recurrence	S/E	3	chr3-178951964-G-C	PIK3CA_p.G1007R	0.341	-	32	96	-	-	-	-	0.0124	12.0265	3	242	Somatic
R10	Recurrence	S/E	3	chr3-41266113-C-G	CTNNB1_p.S37C	-	-	-	-	-	-	-	-	0.0087	8.64013	5	574	Somatic
NU3	No Uterine Recurren	EE	1	chr10-123274774-A-G	FGFR2_p.C154R,FGFR2_p.C382R	0.073	-	10	189	0.0996	438.796	52	522	-	-	-	-	
NU3	No Uterine Recurren	EE	1	chr10-89717769-TA-T	PTEN_p.K267RfsTer9	-	-	-	-	0.0949	111.745	13	137	-	-	-	-	
NU3	No Uterine Recurren	EE	1	chr3-178952084-C-T	PIK3CA_p.H1047Y	0.104	-	16	166	0.1856	800.398	72	388	-	-	-	-	
R11	Recurrence	S/E	3	chr17-7577566-T-C	TP53_p.N239D	-	-	-	-	0.2331	369.46	31	133	-	-	-	-	
R11	Recurrence	S/E	3	chr19-52715971-C-G	PPP2R1A_p.P179R,PPP2R1A_	0.15	-	11	74	0.2049	279.492	25	122	-	-	-	-	
R12	Recurrence	EE	3	chr17-7578508-C-T	TP53_p.C141Y	0.262	-	38	164	0.3134	283.067	21	67	0.0244	63.004	17	696	Somatic
R13	Recurrence	CCC	3	chr19-52715982-C-T	PPP2R1A_p.R183W	-	-	-	-	0.2246	482.485	42	187	0.0266	58.4378	14	526	Somatic
R14	Recurrence	EE	2	chr3-41266125-C-T	CTNNB1_p.T41I	0.22	-	63	306	0.2112	411.028	34	161	-	-	-	-	
NR24	No Recurrence	EE	1	chr10-89692904-C-G	PTEN_p.R130G	0.09	-	7	111	-	-	-	-	-	-	-	-	
NR24	No Recurrence	EE	1	chr3-41266113-C-G	CTNNB1_p.S37C	-	-	-	-	0.1368	284.032	29	212	-	-	-	-	

Supplementary Table 1. All variants detected in tumour and plasma. tumour exome data (AF, QUAL, alt depth, total depth) and panel data for tumour and plasma cfDNA (AF, QUAL, alt depth, total depth, cfDNA variant label)

Supplementary Table 2. Comparison of allele frequency (%) detected by whole exome sequencing (Exome AF) and panel-based sequencing (Tumour AF)

Patient	Gene	Variant	Exome AF(%)	Tumour AF(%)
R1	PIK3CA	P539R	7.1	13.68
R1	PIK3CA	H1047R	5.8	19.41
NR1	PIK3CA	E545K	11.1	11.36
NR1	CTNNB1	S33Y	7.1	12.3
NR3	PIK3CA	Q546H	14.2	26.32
NR4	PTEN	R130G	7.1	ND
NR4	BCOR	N1425S	7.9	ND
NR5	ARID1A	R1772*	5.4	9.68
NR5	MSH6	E544*	17.9	10.06
NR5	POLE	S1906Y	23.2	15.38
NR5	POLE	P286R	14.8	16.06
NR5	PIK3CA	R88Q	12.2	16.44
NR5	PTEN	E7*	18.1	18.05
NR5	TP53	R213*	36.2	39.23
NR7	MSH6	R482Q	12	8.27
NR7	FBXW7	R465H	20	18.91
NR7	PTEN	R233*	24.1	27.69
NR7	ARID1A	D1633fs	19.7	30
NR7	CHEK2	T367MfsTer15	75.9	48.45
NR8	PIK3CA	H1047R	18.8	14.74
NR9	PIK3CA	R88Q	16.7	8.33
NR9	ARID1A	R2158*	17.9	15.38
NR9	PTEN	R130*	19.7	21.29
NR9	FBXW7	R465C	23.2	23.67
NR9	PTEN	K267fs	20.2	ND
NR11	PIK3CA	M1043V	40	36.18
NR11	ARID1A	D1633fs	38.2	38.14
NR11	KMT2D	P647fs	32	ND
NR14	TP53	R248W	25.6	25.2
NR14	PIK3CA	H1047L	42.7	47.76
NR15	PIK3CA	R88Q	18.9	13.21
NR15	PTEN	E7*	18.4	13.7
NR15	CTCF	R339W	13.4	14.12
NR15	POLE	V411L	11.9	15.65
NR15	ARID1A	R1772*	26.4	22.29
NR16	PIK3CA	H1047R	20.6	46.55
NR16	FGFR2	N321D	53.8	78.4
NR17	PIK3CA	Q546K	12.5	17.04
NR17	PTEN	Q17*	37.5	39.86
NR18	PTEN	K267RfsTer9	25.9	26.23
NR18	PPP2R1A	R183W	23	27.46
NR18	ARID1A	D1633fs	24.3	30
NR18	KMT2D	V3089fs	26	ND
R7	PTEN	R130Q	19.6	30.77
R7	KRAS	G12A	15.6	33.96
NR20	PIK3R1	K567E	42.5	51.61
NR20	APC	R876Q	5.1	ND
NR22	KRAS	G12D	14.6	20.38
NR22	PIK3CA	Y1021H	20.8	24.23
NR22	PIK3CA	M1043V	18.1	24.53
NR22	PTEN	R130Q	11.7	16.77
R10	PIK3CA	G1007R	34.1	ND
NU3	FGFR2	C154R	7.3	9.96
NU3	PIK3CA	H1047Y	10.4	18.56
R11	PPP2R1A	P179R	15	20.49
R12	TP53	C141Y	26.2	31.34
R14	CTNNB1	T41I	22	21.12
NR24	PTEN	R130G	9	ND

Standard	Gene	Amino acid change	Expected allelic frequency (%)	5ng AF	10ng AF	20ng AF	5ng 1:10 AF
HD842	APC	p.R2714C	8.00	6.15	5.94	NA	ND
HD842	PIK3CA	p.E542K	1.88	1.15	3.15	NA	ND
HD842	KRAS	p.G12D	2.50	1.06	1.94	NA	ND
HD842	PTEN	p.R130*	2.50	4.06	5.04	NA	ND
HD842	TP53	p.S241F	2.50	1.17	2.41	NA	0.74
HD833	ARID1A	P427P;P44P	37.0%	29.94	NA	30.78	NA
HD833	ARID1A	P1326L;P223L;P943L;P96L	27.5%	29.07	NA	33	NA
HD833	MSH2	NA	96.5%	100	NA	100	NA
HD833	CTNNB1	S26C;S26F;S26Y;S33C;S33F;S33Y	37.0%	32.91	NA	29.14	NA
HD833	CTNNB1	S38del;S45del	12.0%	ND	NA	11.22	NA
HD833	PIK3CA	E545K;E545Q	9.5%	9.39	NA	6.72	NA
HD833	PIK3CA	G914R	28.5%	32.74	NA	30.62	NA
HD833	PIK3CA	H1047L;H1047R	16.0%	18.37	NA	17.28	NA
HD833	FBXW7	NA	35.0%	ND	NA	ND	NA
HD833	PIK3R1	Y73Y	16.0%	24.53	NA	23.24	NA
HD833	APC	Y468Y;Y486Y;Y49Y	25.5%	22.92	NA	25.13	NA
HD833	APC	S1738S;S1756S	33.5%	37.94	NA	33.33	NA
HD833	APC	R2696C;R2696S;R2714C;R2714S	28.5%	33.46	NA	30.47	NA
HD833	PMS2	P159S;P279S;P283S;P335S;P364S;P367S;P418S;P470S	20.0%	16.63	NA	19.33	NA
HD833	KRAS	G13A;G13D;G13V	13.0%	14.83	NA	14.54	NA
HD833	KRAS	G12A;G12D;G12V	6.0%	4.71	NA	5.93	NA
HD833	TP53	NA	32.0%	30.47	NA	27.18	NA

Supplementary Table 3. Hot spot mutations detected in reference standard samples using (AF %) using different template DNA concentrations. Comparison with the expected alleleic frequency(%).

Sample ID	Total Read	Mean Read Length	>= Q20 Bases	Uniformity	Sample Type	Total cfDN	Total Reads	Mean Read Length	>= Q20 Bases	Uniformity	Sample Type
R1	1733082	94	72264597	94.38%	FFPE	20ng	21101229	99	935959793	93.09%	cfDNA
NR1	956626	97	41750567	95.38%	FFPE	9ng	21501387	94	970123004	71.08%	cfDNA
NU1	809652	95	34463267	95.30%	FFPE	20ng	18244474	94	816689934	79.72%	cfDNA
NR2	440885	90	17748660	90.68%	FFPE	16ng	14153607	93	576756716	74.48%	cfDNA
NR3	2839484	97	130348464	94.85%	FFPE	20ng	25952269	88	1094882690	58.11%	cfDNA
NR4	61900	62	1724808	78.81%	FFPE	20ng	25417244	99	1173646028	87.67%	cfDNA
R2	884604	91	35708724	66.40%	FFPE	20ng	13957607	96	616032923	85.67%	cfDNA
NR5	942162	96	41329257	94.43%	FFPE	16ng	14781890	94	628341369	77.70%	cfDNA
NR6	1005048	92	40750431	87.09%	FFPE	8ng	21461656	97	969986310	81.06%	cfDNA
NR7	1511427	95	64016222	94.96%	FFPE	14ng	19881318	97	853554173	84.47%	cfDNA
R3	1120652	93	45043334	89.73%	FFPE	20ng	28392258	95	1298714180	80.18%	cfDNA
NR8	829836	93	34660373	94.34%	FFPE	8ng	19189183	98	910396933	89.56%	cfDNA
NR9	969038	94	40428287	93.79%	FFPE	14ng	17052826	99	820480345	90.40%	cfDNA
NR10	1554557	95	66322310	93.32%	FFPE	9ng	19179057	98	919092639	89.14%	cfDNA
NR11	2558904	95	112460033	94.66%	FFPE	12ng	17667710	98	815113258	91.48%	cfDNA
NR12	1402440	91	55292790	94.45%	FFPE	9ng	16345991	99	749180896	93.39%	cfDNA
NR13	2064786	93	86420322	93.09%	FFPE	20ng	13719277	96	609349848	78.88%	cfDNA
NR14	1883005	96	78530988	94.06%	FFPE	8ng	14471194	97	653533265	92.99%	cfDNA
NR15	1558388	95	62956308	91.23%	FFPE	20ng	11876072	94	525714266	79.06%	cfDNA
NR16	899126	87	33067627	92.25%	FFPE	13ng	18552790	97	838666845	92.51%	cfDNA
NR17	916885	97	39551253	93.47%	FFPE	20ng	22146114	98	986841755	88.34%	cfDNA
R4	2177633	95	88625127	89.22%	FFPE	7ng	15638279	94	705984455	84.77%	cfDNA
R5	1371955	93	54586902	90.67%	FFPE	7ng	18324311	96	826924936	87.75%	cfDNA
NR18	2215166	95	98650271	94.98%	FFPE	11ng	20731964	97	952827051	84.06%	cfDNA
NR19	2448047	95	110869466	95.04%	FFPE	10ng	22769782	99	1070099341	88.41%	cfDNA
NU2	2603375	100	123110674	94.75%	FFPE	19ng	25931352	94	1174963984	74.25%	cfDNA
R6	912733	94	37261623	92.06%	FFPE	20ng	17301013	98	787237781	88.83%	cfDNA
R7	952624	86	34916422	75.37%	FFPE	20ng	19656036	99	885092820	92.45%	cfDNA
NR20	1170253	91	46046701	87.63%	FFPE	7ng	19606323	99	933683292	89.11%	cfDNA
R8	955248	85	36088888	78.43%	FFPE	7ng	16462472	95	684609260	82.60%	cfDNA
NR21	1357697	95	56794636	92.49%	FFPE	18ng	19774182	95	897803883	86.63%	cfDNA
R9	2404469	97	109550548	93.16%	FFPE	15ng	26861201	96	1216409168	78.99%	cfDNA
NR22	2306055	98	106182346	94.35%	FFPE	17ng	20676189	96	978303384	84.50%	cfDNA
NR23	2747547	96	122162505	95.67%	FFPE	20ng	22586901	98	1047891024	83.81%	cfDNA
R10	86142	68	2680295	73.82%	FFPE	15ng	15570478	96	663987663	78.38%	cfDNA
NU3	2457794	98	112563953	95.36%	FFPE	20ng	25076308	94	1144520181	75.57%	cfDNA
R11	1034158	97	43923218	95.24%	FFPE	20ng	21911320	98	971528315	87.05%	cfDNA
R12	620544	92	25643799	94.88%	FFPE	16ng	18948049	96	836101888	83.83%	cfDNA
R13	1559871	87	64213011	91.77%	FFPE	10ng	21219367	98	990913247	85.85%	cfDNA
R14	1036931	96	42996323	96.07%	FFPE	20ng	20154661	98	879369691	86.40%	cfDNA
RN24	1416985	94	58196517	90.16%	FFPE	15ng	16082289	97	712391423	87.28%	cfDNA
NR25	3168866	98	146464030	95.07%	FFPE	9ng	17584005	97	813581033	91.11%	cfDNA

Supplementary Table 4. Panel sequencing metrics and QC data for FFPE tumour DNA and cfDNA. Includes total cfDNA concentration used for sequencing

Parameters	Average	Median	Range
DNA input (ng)	14.74	15.5	5-20
Average Base Coverage Depth (X)	28314.95	28825.5	16433- 39674
Uniformity of Base coverage (%)	83.68	84.76	58.1-93.4
Molecular Coverage (X)	546	472	0-4982
Mapped Reads	19613540	19555545	11,756,779-28,204,569

Supplementary Table 5. Average sequencing metrics across the cfDNA samples

Patient	Locus	Variant	OncoKB Actionability	Evidence Level for Endometrial Cance	OncoKB Link
R1	chr3-178936074-C-G	PIK3CA_p.P539R	Alpelisib + Fulvestrant, Capivasertib + Fulvestrant, Inavolisib + Palbociclib + Fulvestrant	3B	PIK3CA P539R | OncoKB™
NU1	chr17-7578398-G-C	TP53_p.H178D	Prognostic in AML, CLL, Myelopreliferative Neoplasms	-	TP53 H178D | OncoKB™
R1	chr10-123279677-G-C	FGFR2_p.S252W	Erdafitinib, Fexagratinib, Lirafugratinib	4	FGFR2 S252W | OncoKB™
R2	chr10-89692905-G-A	PTEN_p.R130Q	Capivasertib + Fulvestrant	3B	PTEN R130Q | OncoKB™
R2	chr3-178916725-C-T	PIK3CA_p.R38C	Alpelisib + Fulvestrant, Capivasertib + Fulvestrant, Inavolisib + Palbociclib + Fulvestrant	3B	PIK3CA R38C | OncoKB™
R3	chr17-7578190-T-C	TP53_p.Y220C	Rezatapopt	3A	TP53 Y220C | OncoKB™
R4	chr10-89692905-G-T	PTEN_p.R130L	Capivasertib + Fulvestrant	3B	PTEN R130L | OncoKB™
R5	chr17-7577121-G-A	TP53_p.R273C	Prognostic in AML, CLL, Myelopreliferative Neoplasms	-	TP53 R273C | OncoKB™
R5	chr19-52715971-C-G	PPP2R1A_p.P179R	Lunresertib + Camonsertib	3A	PPP2R1A P179R | OncoKB™
R7	chr10-89692905-G-A	PTEN_p.R130Q	Capivasertib + Fulvestrant	3B	PTEN R130Q | OncoKB™
R7	chr10-89717672-C-T	PTEN_p.R233*	Capivasertib + Fulvestrant	3B	PTEN R233* | OncoKB™
R7	chr12-25398284-C-G	KRAS_p.G12A	Binimetinib, Cobimetinib, RMC-6236, Trametinib	4	KRAS G12A | OncoKB™
R7	chr3-178952085-A-T	PIK3CA_p.H1047L	Alpelisib + Fulvestrant, Capivasertib + Fulvestrant, Inavolisib + Palbociclib + Fulvestrant	3B	PIK3CA H1047L | OncoKB™
R7	chr3-41266124-A-G	CTNNB1_p.T41A	Not present		
R10	chr3-178951964-G-C	PIK3CA_p.G1007R	Alpelisib + Fulvestrant, Capivasertib + Fulvestrant, Inavolisib + Palbociclib + Fulvestrant	3B	PIK3CA G1007R | OncoKB™
R10	chr3-41266113-C-G	CTNNB1_p.S37C	Not present		
R12	chr17-7578508-C-T	TP53_p.C141Y	Prognostic in AML, CLL, Myelopreliferative Neoplasms	-	TP53 C141Y | OncoKB™
R13	chr19-52715982-C-T	PPP2R1A_p.R183W	Lunresertib + Camonsertib	3A	PPP2R1A R183W | OncoKB™

Supplementary Table 6. Potentially actionable mutations identified by panel-based sequencing
